# Decrease in cuff pressure during the measurement procedure: an experimental study

**DOI:** 10.1186/2052-0492-2-34

**Published:** 2014-06-02

**Authors:** Shota Asai, Asuka Motoyama, Yuri Matsumoto, Hiroyuki Konami, Hideaki Imanaka, Masaji Nishimura

**Affiliations:** The University of Tokushima Graduate School, Tokushima, 770-0855 Japan; Emergency and Disaster Medicine, Tokushima University Hospital, Tokushima, 770-8503 Japan; Emergency and Critical Care Medicine, Tokushima University Hospital, Tokushima, 770-8503 Japan

**Keywords:** Cuff pressure, Endotracheal tube, Cuff inflator, Cuff shape

## Abstract

**Background:**

To prevent endotracheal tube (ETT)-related complications during mechanical ventilation, ETT cuff pressure should be kept within proper range. In clinical settings, cuff pressure often decreases from target values.

**Methods:**

We performed an experimental study to investigate the effects of measuring devices and endotracheal tubes on change in cuff pressure. We continuously measured cuff pressure by inserting a three-way stopcock in the middle of an ETT pilot balloon system. After adjusting the cuff pressure to 24 cmH_2_O, we disconnected and reconnected each cuff inflator to the inflation valve of the ETT and measured the changes in the cuff pressure. We measured the change in cuff pressure with different ETT sizes, cuff shapes, brands of cuff inflator, and with and without added extension tubes.

**Results:**

The cuff pressure decreased, on average, by 6.6 cmH_2_O (standard deviation 1.9), when connecting the cuff inflator to the pilot balloon. The measured cuff pressure was less than 20 cmH_2_O in 67% of the tests. The cuff pressure decreased more when an extension tube was used. The brand of cuff inflator made no difference to the pressure loss. The cuff pressure decreased more with ETTs of smaller size and with ETTs with pyriform cuffs.

**Conclusions:**

Procedures to connect cuff inflators to inflation valves resulted in the loss of cuff pressure by 6.6 cmH_2_O on average.

## Background

An endotracheal tube (ETT) with a cuff is commonly used during invasive mechanical ventilation. It is recommended to maintain cuff pressure within 20–30 cmH_2_O [1–4]. Excessive cuff pressure increases the risk of tracheal injury and stenosis, and insufficient cuff pressure can result in air leakage, aspiration, and unplanned extubation [4]. A procedure to maintain cuff pressure at appropriate range may reduce cuff leak, aspiration, and tracheal injury. Several factors are known to affect cuff pressure: ETT size, cuff size, initial cuff pressure, measuring devices, and various patient profiles [5]. Despite frequent readjustment, we failed to prevent changes in cuff pressure in critically ill patients [6], and we wondered if there might be a problem related to the measurement procedure. To test a hypothesis that the action of measuring cuff pressure contributed to the loss of pressure, we investigated the changes in cuff pressure during the measurement procedure by using various measuring devices and tracheal tubes with different cuff shapes.

## Methods

**Figure 1:**
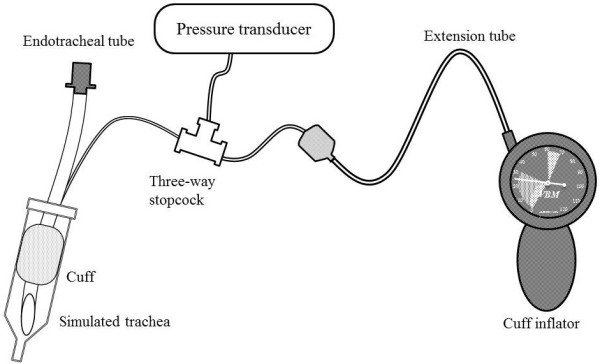
**Experimental setup to investigate endotracheal tube cuff pressure changes during measurement procedure.** A three-way stopcock was fixed in a pilot cuff line of an endotracheal tube.

An experimental setup is demonstrated in Figure 1. For each test, we cut the ETT pilot cuff line in the middle and inserted a three-way stopcock, securely gluing the connecting surfaces around the stopcock so that no leaks were present. Intubation was simulated by placing ETTs in the sheath of a 20-ml syringe (inner diameter, 1.9 cm), because its size was similar to the human trachea (inner diameter, 2.0 cm). A pressure transducer (Medex TranStar MX950, Dublin, OH, USA) was connected to the stopcock, and the cuff pressure was continuously measured with a bedside monitor (Nihon Kohden, BSM-9101, Tokyo, Japan). We connected each cuff inflator to an inflator valve of the ETT and read the value (24 cmH_2_O) displayed on each cuff inflator. We chose such target because pressure reading was only marked every 2 cmH_2_O and it was close to middle of recommended range (20–30 cmH_2_O). After adjusting the cuff pressure to 24 cmH_2_O using a cuff inflator, we waited for equilibrium for about 1 min, disconnected and reconnected the cuff inflator to the inflation valve of the ETT, and calculated the decrease in cuff pressure (Δ*P*_cuff_) when reconnecting the cuff inflator. In a preliminary run, by monitoring the cuff pressure continuously, we had confirmed that the cuff pressure was stable for 30 min without reconnecting the cuff inflator.

We tested three sizes of ETT (internal diameter of 7, 8, and 9 mm) and two cuff shapes—spherical (Blueline, Portex Inc., Keene, NH, USA) and pyriform (TaperGuard Evac, Coviden, Dublin, Ireland)—and both with and without extension tubes, three brands of cuff inflator—Cuff Control Inflator, Sofit (both VBM Medizintechnik GmbH, Sulz am Neckar, Germany), and EndoTest (Rüsch Inc., Duluth, Germany). Because the manufacturer's manual recommended an addition of an extension tube during cuff pressure measurement, we evaluated the effects of the extension tube which was placed between the pilot cuff and the cuff inflator. The same person (SA) repeated the whole test for three times with each combination of ETT size (three kinds), cuff shape (two), and cuff inflator (three), and with or without extension tube (two), resulting in total of 108 measurements. The whole experiment was done in an air-conditioned room (24°C–25°C) of the intensive care unit at daytime. This study was approved by the ethics committee of the Tokushima University Hospital.

### Statistical analysis

Numerical data are shown as mean ± standard deviation, when normally distributed. Comparisons of the decrease in cuff pressure were performed with analysis of variance. When significant differences were observed, multiple comparison testing of means was performed using the paired *t* test with the Bonferroni correction. When not normally distributed, nonparametric tests (Friedman test followed by Wilcoxon signed rank test) were performed. Significance was considered when *p* value was less than 0.05. Statistical analysis was performed using commercial statistical software (SPSS Inc., Chicago, IL, USA).

## Results

**Figure 2:**
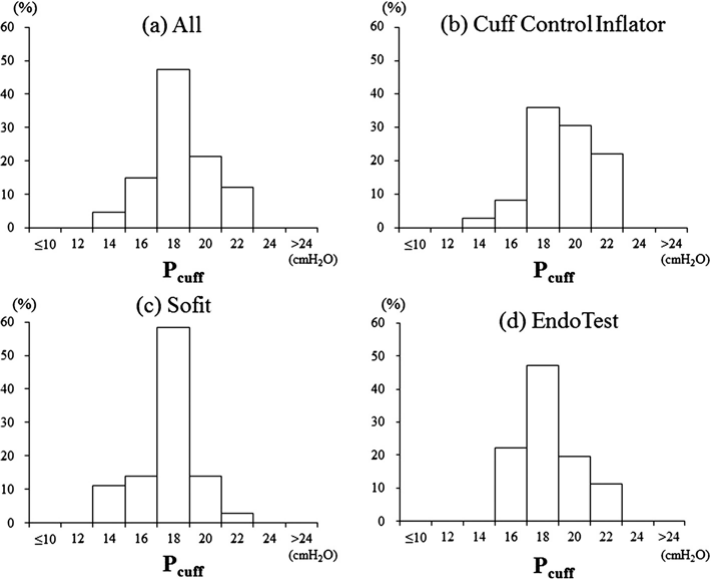
**Cuff pressure distribution. (a)** All cuff inflators. **(b)** Cuff Control Inflator. **(c)** Sofit. **(d)** EndoTest inflators. *Y*-axis shows the percentage of all measurements.

**Figure 3:**
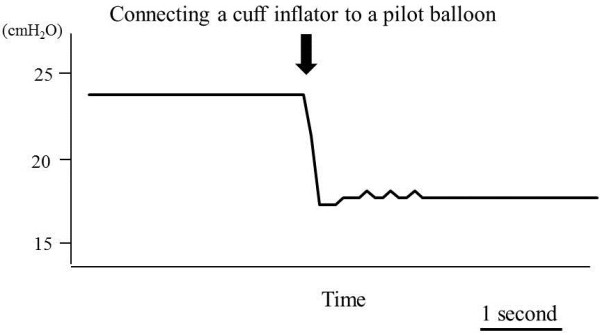
**Representative tracing of cuff pressure during measurement procedure.** Cuff pressure decreased during connection of the cuff inflator to the pilot balloon.

The cuff pressure decreased by 6.6 ± 1.9 cmH_2_O on average with and without extension tubes, when reconnecting the cuff inflator to the pilot balloon. Combining the data from all cuff inflators, the cuff pressure was below 20 cmH_2_O in 67% of the measurements and always less than 22 cmH_2_O (Figure 2a). Three brands of cuff inflators showed similar but different distributions of cuff pressure (Figure 2b,c,d). Figure 3 shows a representative tracing of cuff pressure change.

**Figure 4:**
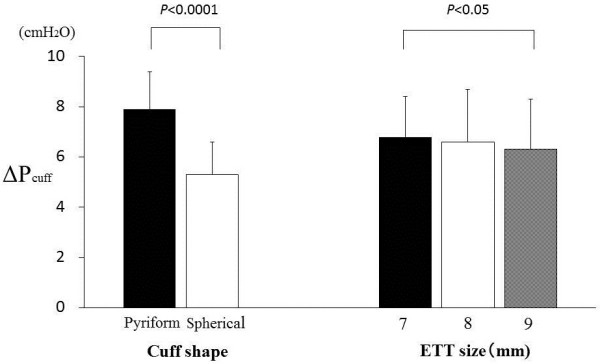
**Effects of cuff shape and endotracheal tube size on cuff pressure change during measurement procedure.**

Effects on Δ*P*_cuff_ values of extension tube, cuff shape, and ETT size are summarized in Table 1. When an extension tube was present, Δ*P*_cuff_ values were significantly larger than when absent (7.5 ± 1.9 cmH_2_O vs. 5.6 ± 1.4 cmH_2_O, *p* < 0.001). There were no significant differences in Δ*P*_cuff_ values among three brands of cuff inflator: Cuff Control Inflator, 7.6 ± 1.7 cmH_2_O; Sofit, 7.8 ± 1.5 cmH_2_O; and EndoTest, 8.2 ± 1.4 cmH_2_O (*p* = 0.50). The Δ*P*_cuff_ values with pyriform cuffs were significantly larger (7.9 ± 1.5 cmH_2_O) than those with spherical cuffs (5.3 ± 1.3 cmH_2_O) (*p* < 0.001, Figure 4). With different ETT sizes, the Δ*P*_cuff_ values were 9 mm, 6.3 ± 2.0 cmH_2_O; 8 mm, 6.6 ± 2.1 cmH_2_O; and 7 mm, 6.8 ± 1.6 cmH_2_O (Figure 4).

**Table 1:** **Effects of extension tube, cuff shape and tube size of endotracheal tubes on cuff pressure drop during measurement procedure**

	With an extension tube			Without an extension tube		
	7 mm	8 mm	9 mm	7 mm	8 mm	9 mm
Pyriform shape	8.9 ± 0.7	9.4 ± 0.5	9.2 ± 0.6	6.5 ± 1.1	6.6 ± 1.1	6.5 ± 0.6
Spherical shape	6.2 ± 1.0	5.9 ± 1.5	5.6 ± 0.8	5.4 ± 1.2	4.4 ± 0.6	4.1 ± 1.0

Mean ± SD (cmH_2_O). The cuff pressure drops are shown for three sizes of endotracheal tubes (internal diameter, 7, 8, and 9 mm), with or without an extension tube, and two kinds of cuff shapes (pyriform and spherical).

## Discussion

In this study, we found that when reconnecting a cuff inflator to a pilot balloon, the cuff pressure decreased by 6.6 ± 1.9 cmH_2_O. This is the first report demonstrating that the procedure for cuff pressure measurement contributes to loss of cuff pressure. The gas pathway within the measuring device needs to have pressure equalization which is proportional to the volume of the pathway. We assume that when connecting the cuff inflator to the pilot balloon, the air compressed inside the cuff escapes into the measurement system, resulting in significant pressure loss, because the pressure in the cuff is 24 cmH_2_O and the pressure in the cuff inflator and extension tube is 0 cmH_2_O. Cuff pressure decreased more when an extension tube was attached. It was likely that the compliance of the measurement system was increased by adding an extension tube, resulting in greater loss of cuff pressure. To avoid these effects, we recommend connecting the cuff inflator directly to the pilot balloon.

Whereas Blanch et al. have reported significant differences among different brands of cuff inflators [7], we found no difference in Δ*P*_cuff_ values among three tested brands of cuff inflator, possibly because the three devices had similar compliance. Blanch et al. speculated that different compressive volumes among the tested cuff inflators resulted in differences in cuff pressure readings. In contrast, prior to this study, we measured the compliance of cuff inflators without an extension tube by injecting the air in 0.5-ml step and found that the compliance was similar (between 0.018 and 0.020 ml/cmH_2_O). Because the greatest drops in cuff pressure were recorded with a combination of pyriform cuffs and 8-mm size ETT (Table 1), it can be assumed that such configurations have the lowest system volume. Nseir et al. have also reported greater changes in cuff pressure with pyriform cuffs than with standard cuffs [8]. Our findings suggest that Δ*P*_cuff_ depends on cuff shape and volume and that when small-volume cuffs are used, cuff pressure requires more careful control.

This study has several limitations. First, we continuously monitored cuff pressure during measurement procedures only in an experimental setup. Because the syringe used in this study has rigid properties and smaller inner diameter than the trachea, the pressure change may be magnified when compared with a flexible tracheal model. Only the same person (SA) performed the connection/disconnection procedure. Further study is needed to confirm whether similar pressure loss occurs with patients in clinical settings during cyclical mechanical ventilation. Second, we did not investigate long-term changes in cuff pressure or the effects of mechanical ventilation on cuff pressure. Monitoring of long-term changes in cuff pressure may also be important in preventing intubation-related complications. Our results suggest that it may be prudent to use devices that intermittently measure and adjust cuff pressure without extension tubes or continuously adjust cuff pressure.

## Conclusion

The cuff pressure dropped by 6.6 cmH_2_O on average when connecting a cuff inflator to an inflation valve of the endotracheal tube. The use of an extension tubing and endotracheal tube with pyriform cuff was associated with larger cuff pressure drop.

## Abbreviations

ETT: endotracheal tube
Δ*P*_cuff_: decrease in cuff pressure.
